# Defining and conceptualizing outcomes for de-implementation: key distinctions from implementation outcomes

**DOI:** 10.1186/s43058-020-00035-3

**Published:** 2020-04-30

**Authors:** Beth Prusaczyk, Taren Swindle, Geoffrey Curran

**Affiliations:** 1 Washington University School of Medicine in St. Louis, 660 S. Euclid Avenue, St. Louis, MO 63110 USA; 2 University of Arkansas for Medical Sciences, 4301 W. Markham Street, Little Rock, AR 77205 USA; 3 Central Arkansas Veterans Healthcare System, 2200 Fort Roots Drive, North Little Rock, AR 72114 USA

**Keywords:** De-implementation, De-adoption, Methods, Outcomes, Measurement

## Abstract

**Background:**

Increasingly, scholars argue that de-implementation is a distinct concept from implementation; factors contributing to stopping a current practice might be distinct from those that encourage adoption of a new one. One such distinction is related to de-implementation *outcomes*. We offer preliminary analysis and guidance on de-implementation outcomes, including how they may differ from or overlap with implementation outcomes, how they may be conceptualized and measured, and how they could be measured in different settings such as clinical care vs. community programs.

**Conceptualization of outcomes:**

We conceptualize each of the outcomes from Proctor and colleagues’ taxonomy of implementation outcomes for de-implementation research. First, we suggest key considerations for researchers assessing de-implementation outcomes, such as considering how the cultural or historical significance to the practice may impact de-implementation success and, as others have stated, the importance of the patient in driving healthcare overuse. Second, we conceptualize de-implementation outcomes, paying attention to a number of factors such as the importance of measuring outcomes not only of the *targeted practice* but of the *de-implementation process* as well. Also, the degree to which a practice should be de-implemented must be distinguished, as well as if there are thresholds that certain outcomes must reach before action is taken. We include a number of examples across all outcomes, both from clinical and community settings, to demonstrate the importance of these considerations. We also discuss how the concepts of health disparities, cultural or community relevance, and altruism impact the assessment of de-implementation outcomes.

**Conclusion:**

We conceptualized existing implementation outcomes within the context of de-implementation, noted where there are similarities and differences to implementation research, and recommended a clear distinction between the target for de-implementation and the strategies used to promote de-implementation. This critical analysis can serve as a building block for others working to understand de-implementation processes and de-implement practices in real-world settings.

Contributions to the literature

- Research on de-implementation is increasingly becoming a focus of implementation researchers. Guidance on how to conceptualize de-implementation outcomes is critical for moving this area forward.
- When conducting de-implementation research, it is important to measure outcomes, such as acceptability, not only of the *targeted practice* but of the *de-implementation process* as well.
- Assessing outcomes at the non-provider-level (i.e., the patient, consumer, or other receiver of the practice) is also critical in de-implementation studies, due to the possible cultural or historical significance to the practice that will impact de-implementation success.

## Introduction

Increasingly, scholars argue that de-implementation is a distinct concept from implementation; factors contributing to stopping a current practice might be distinct from those that encourage adoption of a new one. De-implementation is defined as reducing or stopping the use or delivery of services or practices that are ineffective, unproven, harmful, overused, or inappropriate [1]. Other terms for de-implementation seen in the literature include de-adoption, discontinuation, dis-investment, and even mis-implementation [2]. The rationale for focusing on de-implementation often centers on decreasing healthcare or other costs, reducing unnecessary treatments that may harm or burden the patient or client, or simply replacing an existing practice with a new, more effective one [3–6]. Several research fields have contributed to the de-implementation literature including medical or clinical research, health services, health policy, and of course, implementation science [4, 7–9].

In the field of implementation science, de-implementation research is garnering increasing attention. A search for the keyword “de-implementation” in the journal *Implementation Science* did not return any articles from the journal’s inception in 2006 through 2011. Only 2 articles on the topic were found in 2012. From 2012 to 2016 there were, on average, 2.4 articles (range, 1–4) returned under the keyword search for “de-implementation” per year. However, in 2017 the search returned seven articles related to de-implementation and eight articles in 2018, demonstrating a marked increase in interest in this area. Further, this rudimentary search may be underestimating the number of articles published in the area given the use of alternate terms for de-implementation. In addition to publications, the number of funded grants in the de-implementation area has also increased. Norton and colleagues recently identified 20 grants funded on the topic between 2000 and 2017, with over half of these between 2015 and 2016 (*n* = 11) [1].

Given the groundswell of interest in de-implementation research, guidance on how to optimally conceptualize and conduct this type of research is needed. To date, researchers have offered guidance on topics such as defining de-implementation and determining what practices or interventions should be targeted for de-implementation [5, 6, 10–12]. While these efforts are imperative for moving the area forward, there are still critical gaps that may be inhibiting the advancement of de-implementation research. One such gap is related to de-implementation *outcomes*.

After their analysis of funded de-implementation grants, Norton, Kennedy, and Chambers [1] outline recommendations for raising the profile of de-implementation research. One of these includes the synthesis and conceptualization of de-implementation outcomes [1]. Historically, the field of implementation science has recognized the value of defined outcomes. Proctor and colleagues’ seminal paper outlining eight implementation outcome categories [13] has been cited over 1000 times. This established outcome framework has served as a grounding point for countless research studies and increasingly, newly proposed instruments/measures [14]. It is our goal to offer discussion and preliminary guidance on de-implementation outcomes, including how they may differ from or overlap with implementation outcomes, how they may be conceptualized and measured, and how they could be measured in different settings such as clinical care vs. community programs.

Specifically, we make two key points: First, we suggest key considerations for researchers assessing de-implementation outcomes, such as considering how the cultural or historical significance to the practice may impact de-implementation success and, as others have stated, the importance of the patient in driving healthcare overuse [15]. Second, we conceptualize de-implementation outcomes, paying attention to a number of factors such as the importance of measuring outcomes not only of the *targeted practice* but of the *de-implementation process* as well. Also, the degree to which a practice should be de-implemented must be distinguished, as well as if there are outcome thresholds before action is taken. We include a number of examples across all outcomes, both from clinical and community settings, to demonstrate the importance of these considerations.

### Key considerations

Before discussing our conceptualization of outcomes, an overarching theme that we feel is critical to include in the conceptualization of de-implementation outcomes is the importance of patients, consumers, and community members (henceforth called “stakeholders”), as we argue it is an important factor when conceptualizing de-implementation outcomes. In implementation research, the practice being implemented is usually the most effective, evidence-based, “gold standard” care. However, in de-implementation research, practices are being stopped/removed, and this could be met with a variety of different reactions from stakeholders that may be unique to de-implementation. Below we discuss some of these reactions, the possible reasons for them, and how these reactions relate to de-implementation outcomes.

### Health disparities

Health disparities are differences and/or gaps in the quality of health and healthcare across different groups, as defined by race, ethnicity, socio-economic status, rural/urban location, and other factors [16]. As it relates to de-implementation, the disparity of access to and quality of healthcare between groups means that historically some groups—particularly minority groups—have not achieved health equity and have not always received the best or even the baseline appropriate level of care [17]. As such, it is understandable that some stakeholders might be suspicious or resistant to de-implementation efforts and see it as an attempt to ration/withhold access to evidence-based care or research-based interventions. If a stakeholder perceives they are already not receiving the care they should be, de-implementation efforts could further that feeling and foster distrust in the provider, healthcare system, social service system, etc. Furthermore, this distrust could strain a relationship with stakeholders that may already be fragile due to the historic nature of bias and discrimination in healthcare [18]. In other words, if a stakeholder already suspects they are being discriminated against and not receiving equal access to evidence-based practices, de-implementation efforts could understandably be seen as further evidence of this discrimination. Related to this, black and Hispanic patients are more likely to receive low-value healthcare practices such as imaging for low-back pain and bone density testing [19, 20] and as a result, may be more often presented with de-implementation efforts than their white counterparts.

### Community or cultural relevance

Some practices may have significant relevance or importance to a community or culture, and as a result, stakeholders could question or resist de-implementation efforts. For example, feeding practices of parents have a basis in cultural values and shared experiences of communities. Targeting practices known to be detrimental for removal can stir unintended reactions among stakeholders. For example, in our ongoing work of de-implementation of detrimental feeding practices, adults’ practices, such as encouraging children to eat to be polite (e.g., “no thank you” bites), may undermine children’s ability to self-regulate and tune in to biological signs of hunger. However, this practice maintains social norms about food serving as love and connection. Such realities in the lives of community stakeholders impact the complexity of de-implementation efforts. Further, when asking educators to reduce practices of pressuring children to eat, educators described feeling as if that ignored the reality of the food insecurity they fear for children [21] and what they experience in their own lives [22, 23]. In the clinical setting, the practice of vaginal douching has a strong cultural and community aspect. Some racial and ethnic groups are more likely to douche, and women often learn about the practice from other women in their families or communities [24, 25]. Attempts to discuss the discontinuation of this practice must be done with the understanding that is part of the stakeholder’s community or culture [24, 26]. Therefore, community or culture relevance should be considered when assessing de-implementation outcomes.

### Altruism

For many practices targeted for de-implementation, stakeholder factors are not always prioritized. In the clinical setting, perhaps the most well-known example of this is the unnecessary prescribing of antibiotic medications. While there are some negative consequences to the stakeholder in this instance such as side effects from the unnecessary medication, the greater incentive to stop unnecessary antibiotic prescribing is at the population-level with the threat of antibiotic-resistant bacteria. In this example, the stakeholder—who may be expecting antibiotics because they have received them in the past—may not be motivated to forego the antibiotics because not only do they individually not benefit from it but the risk (e.g., side effects) may seem relatively benign. Explaining and convincing the stakeholder that they should forego antibiotics for the overall good of the population—especially when they personally do not assume considerable risk individually—could prove challenging. This would require stakeholders to act primarily in an altruistic manner and, given the discussion of health equity and culture relevance above, this may be difficult. In fact, research has shown that when de-implementation was explained to patients with an altruistic pitch, patients did not respond favorably and furthermore rated the providers making the altruistic pitch more negatively [27]. In this study, researchers used written vignettes to assess whether patients responded differently when their physicians’ reasoning for withholding low-value care included an altruism component (e.g., taking antibiotics for a sinus infection could result in antibiotic resistance for others). They found no difference in patients’ requests for antibiotics when the altruistic pitch was included but did find that patients rated those physicians who included the altruistic pitch worse than physicians who did not [27]. This is another example of an inherent difference between implementation and de-implementation. The psychological and emotional process stakeholders are faced with when presented with new, best practices (implementation) is likely different from when they are presented with the discontinuation of practices they may expect or desire (de-implementation).

We are not the first to note these issues as they relate to de-implementation [5, 10, 28, 29], but we hope to build on that discussion and examine how this impacts de-implementation research methods, specifically outcome assessment. First, we believe these issues could affect acceptability and appropriateness. Even if all other relevant aspects of a de-implementation study suggest the practice is poised for successful de-implementation, if the stakeholder still finds the practice acceptable or appropriate then de-implementation could be challenging at best and impossible at worst. We often see this type of barrier in the de-implementation of antibiotic prescriptions, where providers have reported that if the patient is insistent that they receive antibiotics, the provider will relent and write the prescription even when the provider is aware of and believes all of the reasons for not writing it [30, 31]. Second, because of the concerns of health equity and minorities related to de-implementation, we believe it is especially important to measure Reach, the representativeness of the participants in a de-implementation study. Ensuring de-implementation efforts do not further health disparities is a critical and ethical issue de-implementation researchers must be aware of and work to avoid. Third, these issues could also affect the feasibility of de-implementing a practice. With the example of antibiotic prescriptions, if the provider is uncomfortable with patients’ resistance or agitation to being told they are not receiving antibiotics, that provider may relent entirely and stop all efforts to reduce writing prescriptions for antibiotics.

In implementation studies, it may not always be necessary to measure outcomes at the stakeholder-level. However, given the unique considerations and issues of de-implementation outlined above, we believe researchers conducting de-implementation studies should prioritize the assessment of key outcomes at the stakeholder level and how these issues affect de-adoption. We understand that stakeholder-level data are often time-consuming and difficult to collect, but if the field desires to understand how and when de-implementation efforts succeed or fail, understanding the role and magnitude of these stakeholder issues is imperative.

## Conceptualizing de-implementation outcomes

We draw from existing outcomes in our conceptualization of de-implementation outcomes [13]. In 2011, Proctor and colleagues published a taxonomy of implementation outcomes that has become widely used across the field [13]. These outcomes include acceptability, adoption, appropriateness, feasibility, fidelity, cost, penetration, and sustainability. We discuss each of these outcomes as they relate to de-implementation.

In addition to the conceptualization of these outcomes for de-implementation purposes, we also drew on each of our substantive areas of expertise to provide illustrative examples of how these outcomes might be conceptualized and measured in different settings including clinical and community settings. These outcomes are summarized in Table 1.

**Table 1:** Conceptualization of de-implementation outcomes

De-implementation outcome	Target of measurement (if applicable)	Definition	Examples	Additional considerations
Acceptability	Practice	The degree to which a *practice* is perceived as *not* agreeable, palatable, or satisfactory	Providers may find 12-step programs for opioid use disorders unacceptable.	It is important to ask the stakeholders of a practice if they find the practice and the idea of stopping that practice acceptable.
	Process	The degree to which the *idea* of stopping a practice is perceived as not agreeable, palatable, or satisfactory	Providers may find stopping the referral of patients to 12-step programs as unacceptable if the providers cannot provide a more acceptable treatment in its place, such as methadone.	
Adoption		The initial decision or action to stop using a practice	A physician makes the decision to stop ordering imaging for patients with low-back pain.	To avoid confusion between implementation and de-implementation studies, we recommend calling this de-adoption.
Appropriateness	Practice	The degree to which a *practice* is perceived to *not* fit, have relevance, or be compatible for a given setting, provider, consumer, issue, or problem.	Emergency room providers may not find smoking cessation as an appropriate practice to provide in the emergency setting.	It is important to ask the end-users of a practice if they find the practice and the idea of stopping that practice appropriate.
	Process	The degree to which the *idea* of stopping a practice is appropriate given a setting, provider, consumer, issue, or problem.	Emergency room providers may not find the idea of stopping smoking cessation as appropriate because they need to provide this to patients to meet hospital quality metrics.	
Cost		The cost of the de-implementation strategies or the costs-saved from stopping the practice.	The costs associated with using local technical assistance as a de-implementation strategy. The costs saved by not ordering routine lipid panels as screening tests for cardiovascular disease.	
Feasibility		The extent to which a practice can be successfully stopped within a given agency or setting	The feasibility of stopping some practices that are required to provide to meet quality improvement metrics.	It is important to understand how stakeholders’ beliefs on acceptability and appropriateness impact providers’ beliefs on the feasibility of de-implementation. It is also important to consider structural, organizational, or procedural barriers to the feasibility of de-implementation.
Fidelity	Practice	The degree to which the entire or whole practice is stopped for the right people and in the right contexts	The number of components of a bundled intervention for ventilated patients that are stopped.	
	Stakeholders	The degree to which the practice is stopped equally, across patients/clients and providers	The number of physicians in the intensive care unit who stop delivering the bundled intervention.	
Penetration		The extent to which the practice is discontinued within a service setting and its subsystems	The number of intensive care units across a large, healthcare system that stops delivering the bundled intervention.	
Sustainability		The extent to which a practice’s discontinuation is maintained	The number of physicians in the intensive care unit who are still no longer delivering the bundled intervention six months after the de-implementation strategies have discontinued.	

We use the following definitions throughout the paper. When we refer to the “practice” we are referring to the intervention, treatment, service, or program that is the target of de-implementation. When we refer to “providers” we are referring to the individual who delivers the practice. This could be physicians or teachers or others. When we refer to “stakeholders” we are referring to non-providers who have a stake in the practice. This includes patients and students but could also include teachers (if they are not providing the practice to students), parents, community members, or others.

### Acceptability

The definition of acceptability as an implementation outcome is “the perception among implementation stakeholders that a given treatment, service, practice, or innovation is agreeable, palatable, or satisfactory.” When considering this outcome for de-implementation research, we identified multiple ways it could be conceptualized and used, depending on the purpose of the research. Acceptability could be measured as the degree to which stakeholders and providers perceive a given practice as *not* agreeable, palatable, or satisfactory. This is the same definition as it would be for implementation research except the focus is on how *un*acceptable the practice is. In de-implementation research, acceptability measured in this way may be used to identify practices that are candidates for de-implementation. If stakeholders or providers no longer find a practice acceptable or to have low acceptability—for whatever reason—then they may be more likely to de-implement that practice.

Another way acceptability could be conceptualized and measured for de-implementation research is the perception among providers or stakeholders that *stopping or de-implementing* the practice, versus the practice itself, is acceptable, palatable, or satisfactory. While nuanced, this definition distinguishes the acceptability of the de-implementation process from the acceptability of the practice targeted for de-implementation. It may seem logical to assume that if a stakeholder or provider finds a practice unacceptable, they would find stopping or de-implementing that practice an acceptable thing to do. However, we can identify situations in which a provider’s or stakeholder’s opinion about the acceptability of a *practice* may not align with their opinion about the acceptability of *stopping that practice*.

For example, sometimes de-implementation occurs simply because a new, better practice is to be implemented and replace the old one (i.e., the evidence has evolved and improved). In those cases, the original practice may still be acceptable to some at the same time stopping that practice is also acceptable. For example, there are multiple “generations” of anti-depressant medications, where new medications are introduced and are generally considered superior to the older medications, for various reasons. Providers may find the old medications acceptable but also find replacing them with the new, better medications acceptable. In other cases, the practice may be acceptable but it was never evidence-based and is now targeted for de-implementation. In communities, for example, some schools have been hesitant to stop the practice of corporal punishment despite experts advocating for alternative practices [32, 33]. Providers or stakeholders, even if they agree the practice is not ideal, hold on to the way things have been done in the past and describe fear for future generations if the practice were to be stopped.

We recommend researchers who set out to measure acceptability in de-implementation research be clear on what question they are attempting to answer and what target of measurement—the practice itself or the idea of de-implementing it—they are assessing for acceptability. In the event they are studying the de-implementation of one practice coupled with the replacement of another, we believe it would be important to assess the acceptability of both practices.

### Adoption

For implementation purposes, the definition of adoption is “the intention, initial decision, or action to try or employ an innovation or evidence-based practice.” The important component of adoption is that it is the *initial* decision to use a practice. When considering how adoption may be conceptualized for de-implementation, it could be conceptualized as the intention or initial decision to stop using a practice. This could be the first time a provider decided, or at least indicated they had the intention, to not order imaging for a patient presenting with low-back pain. Subsequent decisions to not place this order would move beyond adoption, but that initial decision to *try* de-implementation could be considered adoption. We recommend this simply be called de-adoption in the context of de-implementation to avoid any confusion with adoption.

Another conceptualization of de-adoption in de-implementation research is the degree to which the practice is de-implemented equally. This may be at the stakeholder-level (i.e., is the practice being stopped for some patients and not others?) or at the provider-level (i.e., are some providers stopping and not others?). Whether or not a practice is de-adopted equally may depend on if the intent is to stop the practice completely or just reduce its use. It may also depend on if there is a replacement practice or not.

### Appropriateness

The definition of appropriateness is “the perceived fit, relevance, or compatibility of the innovation or evidence-based practice for a given practice setting, provider, or consumer; and/or perceived fit of the innovation to address a particular issue or problem.” To conceptualize appropriateness for de-implementation research, we took a similar approach to acceptability.

Appropriateness could be conceptualized as when the stakeholder or provider perceives the practice to *not* fit, have relevance, or be compatible for a given setting, provider, consumer, issue, or problem. As with acceptability, this measure of appropriateness may be most useful when used to identify candidates for de-implementation. If a stakeholder or provider no longer (or perhaps never did) find the practice relevant or compatible then that practice may be a prime target for de-implementation assuming other relevant factors are present (e.g., the evidence is not strong, it is not cost-effective, etc.). Appropriateness could also be a measure of the appropriateness of the de-implementation *process* in a given practice setting, provider, or consumer; and/or for a particular issue or problem. Like acceptability, it may not always be the case that practices perceived as inappropriate are selected for de-implementation. Therefore, we recommend de-implementation researchers consider if they want to measure the appropriateness of the practice *and/or* the de-implementation of it and to be clear when reporting their findings what their target of measurement was.

### Cost

The cost of an implementation effort varies according to three components: the complexity of (1) the practice, (2) the implementation strategies, and (3) the setting. Given de-implementation inherently means stopping or reducing a practice, and in healthcare often a practice is targeted for de-implementation solely because it is costly or not cost-effective, there may be cost savings and limited new costs with de-implementation alone. Cost savings could be a relevant measure of cost in the context of de-implementation. One could measure the cost of the practice to the healthcare system and to consumers and how much is saved when the practice is stopped.

In other scenarios, a new practice is often being introduced in place of the old one, and therefore, implementation expenses are coupled with de-implementation cost savings. Likewise, strategies to de-implement a practice will likely have associated costs just as implementation strategies would and those could be measured to help determine the true de-implementation costs. We believe that with de-implementation efforts, just as with implementation efforts, the goal is more towards cost-effectiveness as opposed to strict cost savings.

Given that one criterion for de-implementation is the targeting of potentially harmful practices, the cost of de-implementation should be considered in the greater context of the benefit of the services or care provided. In some cases, de-implementation of harmful practices that are free or low-cost may take several years to demonstrate cost-effectiveness given the upfront costs of de-implementation strategies. For example, in the authors’ ongoing work, we are investing considerable costs in strategies (e.g., peer learning collaborative) to support the removal of harmful feeding practices by early care and education teachers. The cost savings of such efforts may not be evident within 1 school year, the typical time frame for studies in the school setting due to logistical concerns. Concerted and similar efforts targeting adults in multiple contexts of a child’s life over several years may be needed to realize cost-effectiveness through improved child health. Researchers should carefully consider the timeframe for assessing cost in de-implementation research.

We also think cost is important to measure in de-implementation because the assumed cost savings associated with stopping or reducing a practice could have unintended consequences for some providers. In rural areas, health care providers are often operating on minimal budgets with little room to absorb a reduction in revenue [34, 35]. If de-implementing a practice significantly reduces their income, that may have an impact on how acceptable, for example, the provider or hospital might view de-implementing that practice. For example, cardiac catheterizations are not always necessary and are costly [36, 37]. However, a rural hospital that is equipped to conduct cardiac catheterizations likely needs to conduct a certain number of them to break even with the costs the hospital incurs to keep the necessary equipment and staff in place to conduct them. If reducing the number of cardiac catheterizations means the hospital could lose revenue and possibly end up closing that lab and no longer being able to offer that service, there is a strong incentive for the hospital to continue to provide this service. That is, cost considerations could drive views on the acceptability of de-implementation.

### Feasibility

In implementation research, feasibility is defined as “the extent to which a new treatment, or an innovation, can be successfully used or carried out within a given agency or setting.” Feasibility is often discussed in relation to appropriateness and acceptability. If a provider finds a practice acceptable and appropriate—they have mentally bought into the practice—they still may find the practice not feasible to adopt or implement due to structural, organizational, or contextual barriers, for example.

To conceptualize feasibility in de-implementation, we must think about the extent to which a practice can be feasibly *stopped* within a given agency or setting. Similar to our thinking of cost, we may assume that because de-implementation is “simply” stopping a behavior, there may not be structural or procedural barriers (e.g., equipment, staffing) to de-implementation. We may assume that once a provider has made the decision to not provide that practice, they can simply proceed with the behavior of stopping, or de-adoption and de-implementation. However, just as with implementation, there could be de-implementation feasibility issues that prevent the stopping of a practice even if providers or stakeholders personally want to stop.

For example, this may be especially relevant in clinical settings in which structural factors related to electronic health records, billing and coding practices, and policies (such as those made by the Centers for Medicare and Medicaid) may present as insurmountable barriers to de-implementation. If a particular practice—a test or procedure—is necessary for quality/safety oversight purposes, then providers may find that practice not feasible to stop even if they find stopping that practice acceptable and appropriate. This exact issue has recently come under scrutiny with the Medicare Merit-based Incentive Payment System (MIPS)/Quality Payment Program (QPP). The MIPS/QPP programs have 271 performance measures that are the basis for the Centers for Medicare and Medicaid Services value-based payment systems. The American College of Physicians assessed the validity (a combination of the impact, appropriateness, feasibility, specificity, and evidence behind the measure) of 86 of the 271 MIPS/QPP that were relevant to ambulatory general internal medicine and found that only 37% were considered valid; while 35% were not valid, and 28% had uncertain validity [38]. Despite more than half of the measures having uncertain or no validity, these measures are still tied to performance measures; therefore, providers may not be able to feasibly stop using them even if they find them unacceptable or inappropriate.

Therefore, while the de-implementation definition of feasibility simply shifts the focus to the feasibility of *stopping* a practice rather than *using or carrying out* a practice, our main recommendation for de-implementation researchers is to not assume that de-implementation is simpler than implementation processes, nor does it represent solely a personal, behavioral decision. Instead, barriers beyond the individual—structural, organizational, contextual, etc.—could make de-implementation not feasible.

### Fidelity

The definition of fidelity is “the degree to which an intervention was implemented as it was prescribed in the original protocol or as it was intended by the program developers.” In the context of de-implementation, fidelity could be conceptualized as the quality of de-implementation. That is, fidelity would be the degree to which the practice is de-implemented for the recommended persons in the recommended situations. For example, de-implementation fidelity would be reflected by the practice being reduced or stopped for the recommended patients. Further, de-implementation strategies would be applied judiciously to ensure a practice would still be available and provided to patients for whom it is appropriate, even if that is a small number of patients.

In addition to the conceptualization of fidelity as the quality of de-implementation, removal of practices that are not evidence-based could have an indirect influence on fidelity to practices that are evidence-based. That is, targets for de-implementation may compete with implementation of evidence-based innovations. In this case, removal of the competing practices may improve fidelity to implementation of the research evidence. In school settings, for example, fidelity to evidence-based trauma-informed care may be difficult in the face of blanket policies about school suspension and/or educator practices that stigmatize students’ mental health concerns. De-implementation of the latter may indirectly improve the use of trauma-informed care practices. Researchers conducting de-implementation work should specify if their fidelity measure is targeted at the quality of the de-implementation, the indirect effect on the quality of implementation of other research evidence, or both [39, 40].

### Penetration

Proctor and colleagues define penetration as “the integration of a practice within a service setting and its subsystems.” The key word in this definition is *subsystems*. Penetration specifically refers to the concept that a practice is spread throughout a setting and does not end with a single entity. For example, a healthcare system may be comprised of hospitals, outpatient care clinics, specialty clinics, and long-term and post-acute care facilities. In a community setting, there are also networks that extend across the country that have federal, state, and local leadership and policies (e.g., Supplemental Nutrition Assistance Program). When measuring the penetration of an implementation effort one would look to see the extent to which the practice was implemented across all of these subsystems within the system. Penetration in a de-implementation study would be the extent to which a practice is discontinued within a service setting and its subsystems. This concept is especially important for de-implementation research because of the ongoing, large-scale campaigns to de-implement low-value care practices, such as the Choosing Wisely campaign [7]. The purpose of these campaigns is to have de-implementation efforts penetrate the system broadly, and thus, penetration would be a key outcome measure for these campaigns.

### Sustainability

For implementation research, sustainability is defined as “the extent to which a newly implemented treatment is maintained or institutionalized within a service setting’s ongoing, stable operations.” For de-implementation research, sustainability may be defined as the extent to which a practice’s discontinuation is maintained. Once a practice has been de-implemented, over time the practice may be re-implemented—intentionally or unintentionally—without continual efforts to maintain and support initial de-implementation. This also relates to feasibility and the possibility that structural, procedural, or societal factors may inhibit or challenge de-implementation efforts in the long-term. Sustainability after initial de-implementation could prove especially challenging if counteracting factors remain strong forces pushing that practice into use.

## Conclusion

We have attempted to provide some guidance to de-implementation researchers on outcome measurement. We conceptualized existing implementation outcomes within the context of de-implementation, noted where there are similarities and differences to implementation research, and recommended a clear distinction between the target for de-implementation and the strategies used to promote de-implementation. We also highlighted the critical role stakeholders play in de-implementation and made recommendations for capturing this role in de-implementation studies.

There is much work to be done to further de-implementation methodology. High-yield areas include linking these outcomes to specific measures, including testing existing implementation measures such as the Acceptability of Intervention Measure, Feasibility of Intervention Measure, and Intervention Appropriateness Measure [14] in de-implementation studies to assess their fit and relevance. For many of the outcomes presented above, our conceptualizations may also be applicable to implementation outcomes, as there is always an interest in measuring the status quo and change from the status quo, and it may be possible to use the same measures for both types of studies. If these and other existing measures are not adequate for adaptation for de-implementation measurement, there is a need to create valid, reliable de-implementation measures. Furthermore, research on whether or not a threshold exists for these outcomes is needed. For example, for how long or to what extent does an intervention need to be found unacceptable before the practice is officially deemed “unacceptable”?

Also, with the conceptualization of these outcomes, it is important to link them with theories, strategies, and mechanisms for de-implementation. As we have discussed, it is reasonable to believe there are distinct differences between implementation and de-implementation. That is, we do not know if the existing theories and strategies for implementation apply in the context of de-implementation. Applying these theories to de-implementation strategies and outcomes is critical to building a solid foundation of de-implementation research. Likewise, we do not fully understand the mechanisms that drive implementation, and by extension, we do not know if these mechanisms are the same for de-implementation. The critical analysis of de-implementation outcomes we offer in this paper can serve as a piece of the methodological puzzle for others working to understand de-implementation processes and de-implement practices in real-world settings.

## Data Availability

Not applicable

## Abbreviations

MIPS: Medicare Merit-based Incentive Payment System
QPP: Quality Payment Program
